# Variability of rRNA Operon Copy Number and Growth Rate Dynamics of *Bacillus* Isolated from an Extremely Oligotrophic Aquatic Ecosystem

**DOI:** 10.3389/fmicb.2015.01486

**Published:** 2016-01-05

**Authors:** Jorge A. Valdivia-Anistro, Luis E. Eguiarte-Fruns, Gabriela Delgado-Sapién, Pedro Márquez-Zacarías, Jaime Gasca-Pineda, Jennifer Learned, James J. Elser, Gabriela Olmedo-Alvarez, Valeria Souza

**Affiliations:** ^1^Laboratorio de Evolución Molecular y Experimental, Departamento de Ecología Evolutiva, Instituto de Ecología, Universidad Nacional Autónoma de MéxicoCoyoacán, Mexico; ^2^Laboratorio de Genómica Bacteriana, Departamento de Microbiología y Parasitología, Facultad de Medicina, Universidad Nacional Autónoma de MéxicoCoyoacán, Mexico; ^3^School of Biology, Georgia Institute of Technology, AtlantaGA, USA; ^4^School of Life Sciences, Arizona State University, TempeAZ, USA; ^5^Laboratorio de Bacteriología Molecular, Departamento de Ingeniería Genética, CINVESTAV – Unidad IrapuatoIrapuato, Mexico

**Keywords:** rRNA operon copies, oligotrophy, bacterial growth, *Bacillus*, Cuatro Ciénegas

## Abstract

The ribosomal RNA (*rrn*) operon is a key suite of genes related to the production of protein synthesis machinery and thus to bacterial growth physiology. Experimental evidence has suggested an intrinsic relationship between the number of copies of this operon and environmental resource availability, especially the availability of phosphorus (P), because bacteria that live in oligotrophic ecosystems usually have few *rrn* operons and a slow growth rate. The Cuatro Ciénegas Basin (CCB) is a complex aquatic ecosystem that contains an unusually high microbial diversity that is able to persist under highly oligotrophic conditions. These environmental conditions impose a variety of strong selective pressures that shape the genome dynamics of their inhabitants. The genus *Bacillus* is one of the most abundant cultivable bacterial groups in the CCB and usually possesses a relatively large number of *rrn* operon copies (6–15 copies). The main goal of this study was to analyze the variation in the number of *rrn* operon copies of *Bacillus* in the CCB and to assess their growth-related properties as well as their stoichiometric balance (N and P content). We defined 18 phylogenetic groups within the *Bacilli* clade and documented a range of from six to 14 copies of the *rrn* operon. The growth dynamic of these *Bacilli* was heterogeneous and did not show a direct relation to the number of operon copies. Physiologically, our results were not consistent with the Growth Rate Hypothesis, since the copies of the *rrn* operon were decoupled from growth rate. However, we speculate that the diversity of the growth properties of these *Bacilli* as well as the low P content of their cells in an ample range of *rrn* copy number is an adaptive response to oligotrophy of the CCB and could represent an ecological mechanism that allows these taxa to coexist. These findings increase the knowledge of the variability in the number of copies of the *rrn* operon in the genus *Bacillus* and give insights about the physiology of this bacterial group under extreme oligotrophic conditions.

## Introduction

Population genetics is the most direct tool for use in understanding adaptation to the ecological challenges imposed upon microbial communities by the environment (Whitaker et al., 2003; Xu, 2006; Spencer et al., 2008). Functional traits can aid in the study of population genetics, because they help to define species in terms of their ecological roles, such as how they use environmental resources or how they interact with other species (McGill et al., 2006; Hughes et al., 2008). These functional traits are often considered to be ecological strategies because they are useful in understanding why certain bacteria live in a particular environment and how they respond to environmental challenges (Green et al., 2008).

The ribosomal RNA operon (*rrn* hereafter) is the key genetic structure for protein synthesis and thus a functional trait related to bacterial life history (Stevenson and Schmidt, 2004). Ecologically, the *rrn* operon has been related with the bacterial capacity to respond to changes in environmental conditions (Codon et al., 1995; Prüβ et al., 1999; Green et al., 2008). In particular, the variation in the number of copies of the *rrn* operon has been considered an ecological strategy related to resource availability, with physiological implications associated with bacterial growth rate and fitness (Klappenbach et al., 2000; Shrestha et al., 2007). The *rrn* operon is comprised of three genes (5S, 16S, and 23S rDNA) and its copy number varies from 1 to 15 among bacterial genomes (Klappenbach et al., 2001; Acinas et al., 2004; Stoddard et al., 2015) and even more dramatically among eukaryotes (Elser et al., 2000). Experimentally, it has been shown that deletions of one or more copies of the *rrn* operon have a considerable impact on growth rate, affecting various stress-response mechanisms (Nanamiya et al., 2010; Yano et al., 2013). Hence, it has been suggested that the multiplicity of the *rrn* operon is a potential mechanism for adaptation to different environmental conditions (Elser et al., 2000; Green et al., 2008). In general terms, bacteria that possess more *rrn* operon copies may cope better with fluctuating nutrient inputs than bacteria with fewer *rrn* operon copies, which tend to live in environments where nutrients are scarce (Klappenbach et al., 2001; Elser, 2003; Jeyasingh and Weider, 2007). Moreover, the relationship between *rrn* operon copy number and the bacterial biotic potential for the cellular allocation of key resources could be analogous to the ecological strategies described in other macro-biota (r- and K-strategies), (Pianka, 1970; Elser et al., 2000; Dethlefsen and Schmidt, 2007; Shrestha et al., 2007; Lipowsky et al., 2012).

*Bacillus* is a genus that is well-known because of its ecological versatility (Feldgarden et al., 2003). The fact that it can sporulate increases its long-range dispersal and allows it to explore diverse environments (Nanamiya et al., 2010; Yano et al., 2013). Coincidentally, *Bacilli* have a relatively high number of *rrn* operon copies per genome, ranging from six to 15 (*rrn*DB, Stoddard et al., 2015), a degree of variation that may aid in this lifestyle strategy of colonizing new environments and provide great adaptability in response to stress, as well as being able to uptake a wide variety of resources (Feldgarden et al., 2003; Connor et al., 2010). If the new environment is rich in phosphorus (P), high *rrn* operon copy number may be favored because the rich P supply could then support the rapid production of P-rich *rrn* to meet the protein demands of rapid growth, as stated by the “Growth Rate Hypothesis” (GRH), a core idea within the theory of biological stoichiometry (Elser et al., 2000; Elser, 2006). However, in environments with low phosphorus availability, multiple *rrn* operon copies could represent a competitive cost if a high *rrn* operon copy number leads to the over-production of P-rich *rrn* (Sterner and Elser, 2002; Maciá, 2005; Jeyasingh and Weider, 2007). Indeed, aquatic bacteria isolated from oligotrophic environments usually have low *rrn* operon copy numbers (Fegatella et al., 1998; Strehl et al., 1999; Lauro et al., 2009), as well as various other adaptations to decrease cellular phosphorus demand (Cavicchioli et al., 2003; Alcaraz et al., 2008; Martiny et al., 2009; Van Mooy et al., 2009). Thus, it has been proposed that there is a connection between the number of *rrn* operon copies and environmental P availability (Elser et al., 2000; Weider et al., 2005; Jeyasingh and Weider, 2007). However, to our knowledge, we lack extensive studies that document this variation in *rrn* operon copy number and other associated ecological strategies employed by bacteria coexisting in oligotrophic environments, especially those characterized by severe P limitation.

The aims of this study were to describe *rrn* operon copy number variation in different lineages of *Bacillus* strains isolated from an extremely oligotrophic ecosystem and to analyze the possible association between the copy number and its physiological implications for growth rate and chemical composition (P content and N:P stoichiometry). Severe P limitation is considered a primary selective pressure that drives bacterial evolution in this environment (Souza et al., 2008, 2012). For example, we have previously reported on an endemic and moderately halophilic *Bacillus* (type strain of *B. coahuilensis*: m4-4 = NRRL B-41737^T^), (Cerritos et al., 2008) that has a typical number of *rrn* copies (nine) but also clear adaptations to the extreme oligotrophic conditions, including a small genome (3.5 Mb), a diversity of phosphate acquisition genes (Moreno-Letelier et al., 2011), and a cellular membrane composed of sulfolipids (Alcaraz et al., 2008). Similar adaptations to low P levels have been reported only from oligotrophic marine cyanobacteria with low *rrn* copy numbers (Cavicchioli et al., 2003; Lauro et al., 2009; Martiny et al., 2009; Van Mooy et al., 2009). Hence, the present study represents the first attempt to link biological stoichiometry to *Bacillus* diversity and *rrn* operon copy number, as well as the first report in which the numbers of *rrn* copies are analyzed in several members of this genus that coexist in the same habitats.

## Materials and Methods

### Site Description and Selection of *Bacillus* Strains

**FIGURE 1:**
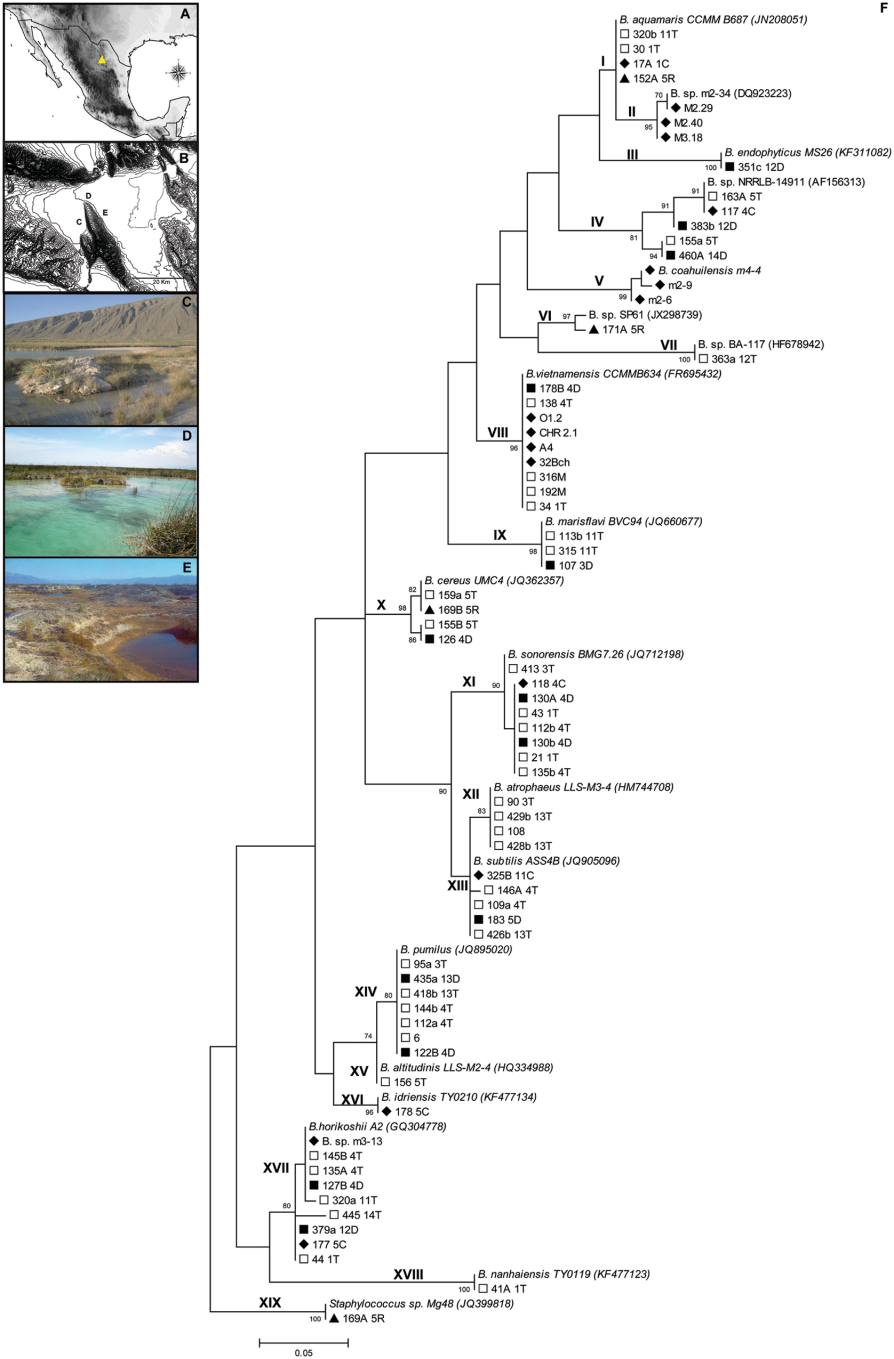
**The Cuatro Ciénegas Basin (CCB) in the Chihuahuan Desert, in northeastern México **(A,B)** and the sites where *Bacillus* strains were previously isolated.** **(C)** The Churince system, **(D)** Pozas Rojas (Los Hundidos), and **(E)** Río Mesquites. **(F)** Maximum-Likelihood (ML) tree of the 19 phylogenetic groups identified using the 5′ HV region of the 16S rDNA. Bootstrap values higher than 70% are shown. Symbols represent the sample type of isolation: 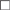 = Top section of sediment, 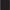 = Bottom section of sediment, 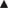 = Water sediment adjacent to a plant, and 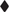 = Water.

The Cuatro Ciénegas Basin (CCB hereafter) is a hydrologic system in the Chihuahuan Desert in northeastern México (Souza et al., 2006), (**Figure 1A**, yellow triangle). This basin represents an oasis with a high microbial diversity in extremely oligotrophic conditions (<1 μmol PO_4_^3-^; Peimbert et al., 2012; Souza et al., 2012). Interestingly, ca. 50% of the bacterial communities in the CCB are most closely related to marine relatives (Souza et al., 2006). Isolates related to the genus *Bacillus* were identified from samples collected at various sites in the CCB during 15 years of field work (Souza et al., 2006; Alcaraz et al., 2008, 2010; Cerritos et al., 2010; Pérez-Gutiérrez et al., 2013), (**Figure 1B**).

Our isolates are from three primary sampling sites within the basin: (i) the Churince site consists of a freshwater spring that connects to an intermediate shallow pond via a small stream and eventually terminates in a shallow desiccated lagoon (**Figure 1C**); (ii) the Río Mesquites is a stable system composed of a river and some lateral desiccated ponds (**Figure 1D**) with low nutrient concentrations and highly imbalanced C:N:P ratios [C:N:P, 900:150:1 (molar); Souza et al., 2012]; and (iii) the Pozas Rojas site (**Figure 1E**) is located in a system called Los Hundidos and consists of a shallow lake and nine to 12 small semi-permanent ponds with strongly fluctuating conditions (high salinity and temperature in summer, both decrease in winter).

The *Bacillus* strains isolated from the CCB are part of a larger collection (several thousands of isolates) of microbes that is maintained at the Molecular Evolution and Experimental Laboratory at the Instituto de Ecología, UNAM (Valeria Souza) and at the Molecular Bacteriology Laboratory in the CINVESTAV, Irapuato (Gabriela Olmedo); cultures are available upon request. We selected 71 of these isolates and classified them according to the site of isolation and sample type (plant root, sediment, or water). Sixty-seven *Bacillus* isolates were sampled from Churince, one was from Río Mesquites and three were from Pozas Rojas.

### Phylogenetic Analysis

To obtain biomass for DNA extraction, *Bacillus* isolates were grown in the standard medium used for their isolation in the field (Marine agar, Difco^TM^ 2216). Genomic DNA extractions were performed using the QIAmp^®^ DNA Mini Kit (USA), according to the manufacturer’s instructions. The 5′ hypervariant (HV) region of the 16S rDNA was amplified (275 bp; 70–344 position), following Goto et al., (2000). This region has a high level of conservation and is useful for the clustering of *Bacillus* species. The polymerase chain reaction (PCR) products were confirmed via 1.5% agarose gel electrophoresis. The sequencing of the HV region was performed by the High Throughput Genomics Center (htSEQ), University of Washington (USA), and compared with the GenBank database using BLAST (NCBI). Sequences were aligned using CLUSTAL W (Thompson et al., 2002), and a maximum-likelihood tree was constructed using MEGA5, with a bootstrap of 1000 replicates (Tamura et al., 2011). The sequences of the 5′ HV region of the 16S rDNA were submitted to GenBank with the following accession numbers: KT781592–KT781661.

### I-*CeuI* Cleavage Map of the *Bacillus* Strains

The I-*Ceu*I (*Ceu*I hereafter) restriction endonuclease recognizes a 26-bp sequence from position 1911–1936 of the 23S rRNA gene in *rrn* operons, with the number of *Ceu*I (New England Biolabs) fragments usually representing the number of *rrn* operons. Pulsed-field gel electrophoresis (PFGE) was used to construct the *rrn* profile of the chromosome from the *Bacillus* isolates. Bacterial genomic DNA from *Salmonella enterica* serovar Typhimurium LT2 cleaved with *Ceu*I and the 0.1–200 kb Sigma Plus Marker were used as molecular weight markers. Because the size of *S. enterica* Typhimurium LT2 identifying fragments had been determined previously (Liu et al., 1993), their inclusion improved the precision of the band-size estimation.

### Preparation and Digestion of Genomic DNA in Agarose Blocks

*Bacillus* isolates were cultured aerobically in Difco^TM^ Marine Broth 2216 (BD & Co.) and incubated overnight at 35°C. The genomic DNA of each strain was prepared in agarose blocks using a previously described method, with some modifications (Nakasone et al., 2000; Delgado et al., 2013). Two processes of incubation in proteinase K solution (12 h at 37°C) were carried out to increase the purity of the DNA. Agarose blocks were pre-incubated in 1X NEBuffer 4 (New England Biolabs) for 30 min at 4°C. Finally, the digestion of the genomic DNA was achieved with 100 μl fresh 1X NEBuffer 4 containing 15 U of I-*CeuI* restriction enzyme, and it was incubated overnight at 37°C.

### Pulsed-Field Gel Electrophoresis (PFGE) and DNA Fragments Transfer

The *Ceu*I fragments were separated by a CHEF-DR II electrophoresis system (Bio-Rad). Electrophoresis was performed on a 1% agarose (Seakem Gold agarose, BioWhittaker Molecular Applications) gel and 0.5X TBE buffer (Bio-Rad) at 11°C. The electrophoresis conditions were divided into two stages to separate the DNA fragments of various sizes: First stage, pulse time ramped from 6.75 s to 2 min for 20 h at 4 V cm^-1^ and in a second stage, pulse time ramped from 0.22 to 5.10 s for 15 h at 6 V cm^-1^.

The agarose gels were radiated with UV light for 1 min in a UV Crosslinker (UVP) to fix the DNA. The gels were washed in 250 mM HCl solution for 15 min with moderate shaking. Next, the gels were washed in denaturing buffer (1.5 M NaCl, 0.5 M NaOH) for 2 h and later washed in a neutralization buffer (0.5 M Tris/HCl, 1.5 M NaCl; pH 8.0) for 2 h. The DNA fragments were then transferred onto N+nylon membrane (Amersham Biosciences) via Southern blotting as described previously (Sambrook et al., 1989). Finally, the membrane was radiated with UV light to fix the DNA (1 min; UV Crosslinker, UVP).

### Preparation of DNA Probes and Hybridization

The *rrn* profiles of *Bacillus* isolates were validated by probing the Southern blotting membranes with PCR products of the 16S and 23S *rrn* operon from the *Bacillus horikoshii* ATCC 700161 strain. The primer sets used to amplify the *rrs* gene were designed using the 5′ HV region (described above), and an internal region of the *rrl* gene was designed from the 2283 to 2696 position (23S_3_) of the *rrn*. Then, the 23S_3_ region (413 bp) was amplified using the forward primer F23S_3_ 5′-ACG GAG GCG CCC AAA GGT T-3′ and the reverse primer R23S_3_ 5′-CCA GCG GTG CGT CCA TCC-3′. The primer set used to amplify the 23S_3_, was designed based on previously sequenced genomes using the Primer Select program of the DNASTAR Lasergene 7 package (DNASTAR, Inc., Madison, WI, USA).

The PCR amplification conditions were as follows: 95°C for 5 min for the initial denaturation, 30 cycles of denaturation at 95°C for 40 s, annealing at 60°C for 40 s, an extension of 1 min at 72°C and a final extension of 5 min at 72°C (Gene Amp, PCR System 9700). The presence and size of PCR products were subsequently confirmed via 1.5% agarose gel electrophoresis. The PCR products were purified with the PCR Clean-up Gel Extraction Kit (Macherey–Nagel products) and then DIG-labeled using the random primer method of the DIG High Prime DNA Labeling system (Roche). The membrane was incubated in 10 ml hybridization solution (DIG Easy Hyb buffer). Incubation was carried out at 58°C with constant, gentle shaking for 1 h. The labeled probe was then added to fresh hybridization solution and hybridization was carried out overnight at 58°C with constant and gentle shaking. The membrane was exposed to X-ray film after being washed at high astringency (64°C).

### Growth Parameter Estimations

Genotypes of *Bacillus* with different numbers of copies of the *rrn* operon were chosen from among the groups described in the phylogenetic analysis. Prior to growth parameter estimation, all the strains were pre-cultured in fresh marine broth for 24 h to homogenize their metabolic condition. All cultures were incubated at 35°C, the maximum water temperature during summer at the CCB (Pérez-Gutiérrez et al., 2013), with shaking at 150 rpm. Additionally, experiments were carried out for nutritional conditions similar to CCB; for this, we inoculated the strains into sterile water collected from the Churince field site but supplemented with tryptone (5 g per liter; Bacto^TM^ Tryptone, BD and Company; hereafter, CCBwt) and incubated for 12 h (overnight), (**Supplementary Figure S1A**).

The growth parameters were then determined using the previously described overnight culture. Three new 50 ml flask of fresh CCBwt medium were inoculated to reach an optical density of 0.05 (600 nm wavelength; BioPhotometer Plus, Eppendorf), which corresponded to ∼10^7^ colony-forming units (CFU) ml^-1^. CFU counts were made taking at least seven samples distributed through a period of 12 h to cover all the phases of the growth curve, the samples were diluted appropriately in 0.85% NaCl to perform a plate count analysis.

We estimated the lag phase period (λ; *units: hours*), the tangential growth rate (*G*_tan_*; units: cells/h*), and maximal biomass reached [*A; units*: *Ln*(*CFU/CFU*_(t__ =__0)_)] from our data using a non-linear regression (CurveExpert Professional 2.0.3 software) to fit a Gompertz equation according to Zwietering et al. (1990), (**Supplementary Figure S1B**). We obtained the final parameters from the predicted curve, defining *G*_tan_ as the tangent of the inflection point of the curve, λ as the X intercept of the tangential line through the inflection point (where the *X*-axis is time), and *A* as the Y-value of the asymptote [where the *Y*-axis is *Ln*(*CFU/CFU*_(t__ =__0)_)] for plate-count assays. In addition, we estimated the maximum specific growth rate (μ_max_; units: hours^-1^) as follows:

$$\mu_{\max}=In\left(N_{e}-N_{0}\right)/\left(t_{e}-t_{0}\right)$$

and the bacterial doubling (generation) time as follows:

$$t_{d}=In\,\;\,2/\mu_{\max}$$

*N*_0_ and *N*_e_ are the cell densities reached at the beginning and at the end of the exponential phase, respectively, while *t*_0_ and *t*_e_ are the times (h) at which the exponential phase started and ended, respectively.

### Cell Contents of Carbon (C), Nitrogen (N), and Phosphorus (P) During the Exponential Phase

The samples of bacterial biomass were harvested in the exponential phase during the determination of growth dynamics. Biomass samples were spin in a centrifuge for the removal of the growth medium. To avoid the influence of remains of the growth medium in the elemental composition analysis, the biomass samples were washed three times with 250 μl water (Mili-Q)^®^ and after of each washing, supernatant was removed by centrifuge. Finally, the biomass samples were vacuum dried to be shipped frozen to Arizona State University for analysis.

At ASU total phosphorus content was measured using a modified ascorbic acid method with persulfate digestion (APHA, 2005). The dried biomass samples were weighed and treated with a potassium persulfate and sulfuric acid solution and then autoclaved for 30 min at 121°C and 15–20 psi. The samples were allowed to cool and then neutralized before the addition of the color reagent. After 30 min, the absorbance was read on a spectrophotometer at 880 nm. The samples were analyzed with a triplicate standard curve and triplicate NIST reference material.

The total carbon and nitrogen content was measured via combustion in a Perkin Elmer model 2400 elemental analyzer. The samples were combusted at 1760°C. Elemental detection was conducted via a thermal conductivity detector.

The C, N, and P data were expressed as percentages of dry mass and referred to as “C content,” “N content,” and “P content,” respectively (**Supplementary Table S2**).

### Statistical Analysis

All statistical analyses, including the estimation of Pearson correlation coefficients (*r*) and principal component analysis (PCA) were performed with the *R* Statistic program Version 3.3.1 (24-07-10).

## Results

### Identification and Clustering of *Bacillus* Isolates from the CCB

The 71 isolates used for this study were clustered into 19 phylogenetic groups, forming a large “marine” cluster (30 isolates; i.e., formed by strains with marine affinities). Most of the lineages include representatives from a variety of habitats (e.g., soil, water, sediment); however, some “marine” groups included strains only sampled from water, while the *B. atrophaeus* lineage was only found in the top layers of sediment. Strains related to *B. pumilus* were also only found in sediment (**Figure 1F**).

The “marine” cluster is composed of nine phylogenetic groups (arbitrarily numbered I to IX) composed of *Bacillus* strains isolated mostly from CCB aquatic samples, such as *B.* sp. m2-34 (group II) and *B. coahuilensis* (group V). In addition, three CCB phylogenetic groups of *Bacillus* were related to type strains isolated from water: *B. endophyticus* (group III), related to a pollutant-degrading strain isolated from industrial eﬄuent, as well as *B.* sp. NRRLB-14911 (group IV) and *B. marisflavi* (group IX), both isolated from seawater. The *B. aquamaris* (group I), *B.* sp. SP61 (group VI), and *B. vietnamensis* (group VIII) groups are related to strains isolated from hypersaline environments (salterns and a microbial mat). Finally, group VII is related to a strain isolated from a biofilm of a lake (*B.* sp. BA-117). The small *B. cereus* cluster is related to type strains isolated from marine sediments (group X). The *B. subtilis* cluster (group XIII), was similar to soil type *Bacillus.* Group XI is most closely related to *B. sonorensis* isolated from the soil of the Sonoran desert. Organisms in group XII are related to a *B. atrophaeus* strain isolated from soil and water samples, and group XIII is related to a *B. subtilis* strain from marine samples, although this is a well-known cosmopolitan species. Strains related to a *B. altitudinis* strain (group XV), were present in soil and water samples; *B. idriensis* isolates (group XVI), were related to strains from soil samples with halotolerance. Finally, the *B. horikoshii* cluster (group XVII) has as a representative a strain isolated from a fish pond as well as a lineage related to a halotolerant *B. nanhaiensis* (group XVIII), isolated from a non-saline soil sample. Unexpectedly, the *169A 5R* isolate was closely related to the strain *Staphylococcus* sp. Mg48 (JQ399818), isolated from a saline lake (group XIX), but it is not unusual to isolate a *Staphylococcus* strain when aiming to select *Bacillus*.

### Phylogenetic Variability of the *rrn* Operon Copy Number

**FIGURE 2:**
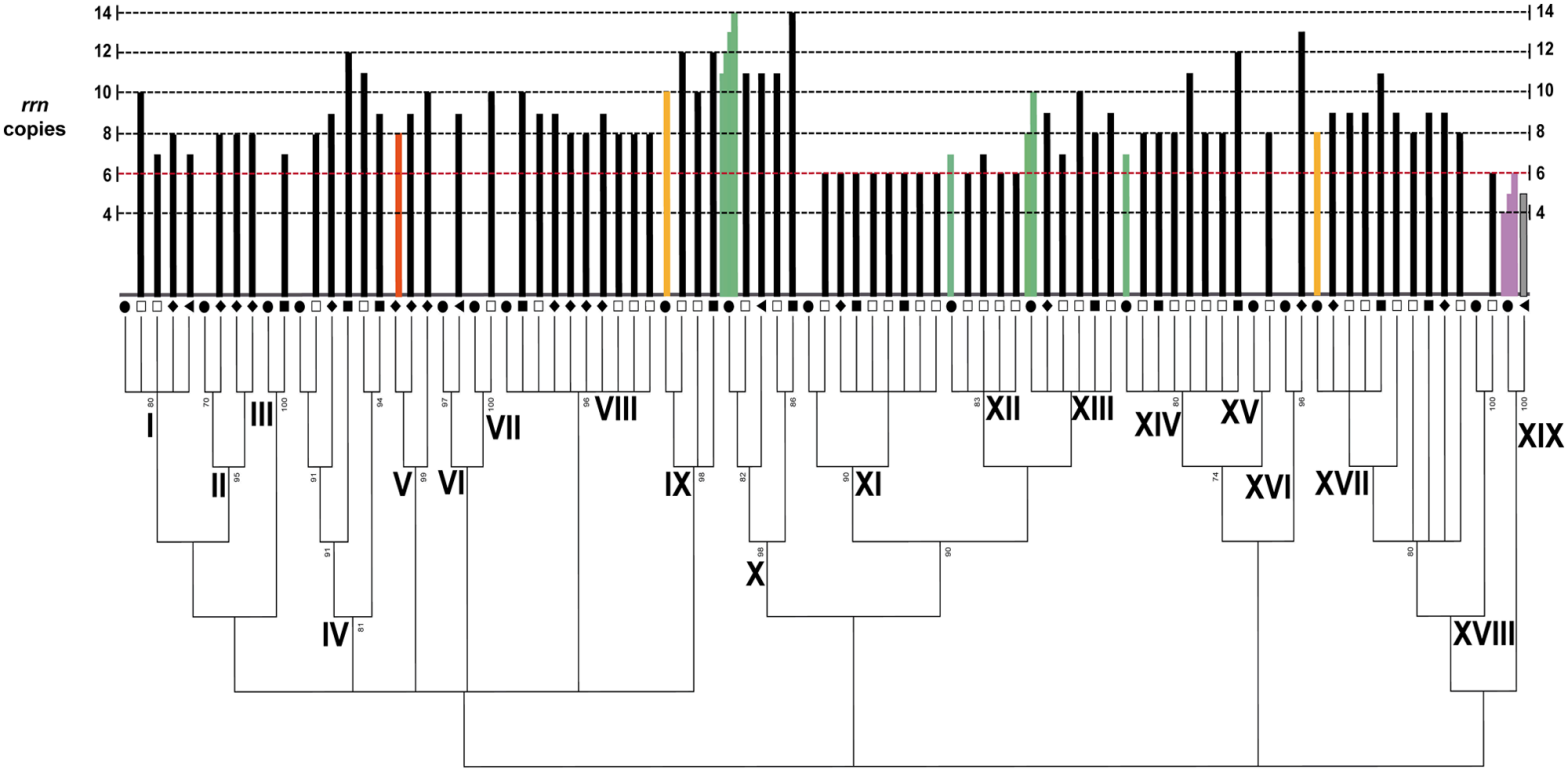
**Variability of the rRNA operon copy number in the *Bacillus* diversity from the CCB.** ML tree of the 5′ HV region of the 16S rDNA with a 50% of cut-off value. The black circles represent the type strain of *Bacillus* for every phylogenetic group. The squares, triangles and rhomboids symbols correspond to the environment as described in **Figure 1.** The dotted red line represents the low number of the *rrn* operon in *Bacillus* according to the *rrn*DB. The black bars represent the number of operon copies in the isolates from the CCB, and the green bars represent the number of copies in *Bacillus* species reported in the *rrn*DB. Gray bar represents the number of copies in the isolate related to the genus *Staphylococcus*. The light purple bars represent the number of operon copies in different *Staphylococcus* species analyzed by the *rrn*DB. The orange bar represents the number of copies quantified in the type strain of *B. coahuilensis* (m4-4 = NRRL B-41737^T^), group V. The yellow bars represent the number of copies in the type strains of *B. marisflavi* (JCM 11544 = KCCM 41588) and *B. horikoshii* (ATTCC 700161), groups IX and XVII, respectively.

The *rrn* operon copy number was determined for every isolate described above in the maximum-likelihood tree via PFGE and hybridization analyses (**Figure 2** and **Supplementary Figure S2**). To obtain a benchmark for the *rrn* copy number in the *Bacillus* diversity from the CCB, we analyzed the type strain of *B. coahuilensis* (m4-4 = NRRL B-41737^T^) that was isolated from the Churince site (Cerritos et al., 2008) and that has already been sequenced (Alcaraz et al., 2008). After genomic digestion and hybridization analysis, eight *rrn* operons were quantified (**Figure 2** and **Supplementary Figure S2**; group V). The 70 strains of *Bacillus* from the CCB showed a range of between six and 14 *rrn* operon copies (**Figure 2** and **Supplementary Figure S2**). Some groups showed intraspecific variation from one to four copies. Interestingly, we quantified only six copies of this operon in some phylogenetic groups, such as *B. sonorensis* (XI), *B. atrophaeus* (XII), and *B. nanhaiensis* (XVIII); the lowest number of copies quantified in other strains of the genus (*rrn*DB, Stoddard et al., 2015). The highest number of copies was observed in the *B. cereus* group (X) (14 copies).

To further increase knowledge about the number of copies of the *rrn* operon in the genus *Bacillus*, we analyzed two type strains similar to those observed in the CCB, *B. marisflavi* (JCM 11544 = KCCM 41588), and *B. horikoshii* (ATTCC 700161). The genomes of these species have not yet been sequenced and the number of copies of this functional gene is unknown. The *B. marisflavi* and *B. horikoshii* type strains showed ten and eight copies of the *rrn*, respectively (**Figure 2** and **Supplementary Figure S2**, groups IX and XVII). Thus, the number of copies quantified in these *Bacillus* type species was similar to the number described in the strains isolated from the CCB (**Figure 2**).

Homogeneity in *rrn* operon copy number was observed in the *B.* sp. m2-34 (II; eight copies) and *B. sonorensis* (XI; six copies) groups. However, considerable heterogeneity and intraspecific variation were observed in several other groups: *B. aquamaris* (I; seven to nine copies), *B.* sp. NRRLB-14911 (IV; eight, nine, eleven and twelve copies), *B. vietnamiensis* (VIII; eight to ten copies), *B subtilis* (XIII; seven to eleven copies), *B. pumilus* (XIV; eight, eleven, and twelve copies) and *B. horikoshii* (XVII; eight, nine and eleven copies). In addition, the phylogenetic groups composed of only one isolate showed different numbers of *rrn* operon copies: *B. endophyticus* (III; seven copies), *B*. sp. SP61 (VI; ten copies), *B.* sp. BA-117 (VII; nine copies), *B. altitudinis* (XV; eight copies), *B. idriensis* (XVI; 13 copies) and *B. nanhaiensis* (XVIII; six copies; **Figure 2**). The *Staphylococcus* isolate had five copies of the *rrn*.

### Growth Parameters

Growth parameters were estimated for a subsample of 15 *Bacillus* isolates from the CCB representative of the phylogenetic diversity present and the range of *rrn* operon copy numbers observed. We also characterized the isolate related to the *Staphylococcus* genus (**Table 1**). Not surprisingly, given the large diversity in this genus within the CCB, the results show a high heterogeneity in the growth parameters estimated. In agreement with these results, the lag phase period of these *Bacilli* is variable and it is not related with their growth rate (μ_max_; **Supplementary Figure S3A**). In addition, the maximum biomass reached was correlated with the maximum specific growth rate (μ_max_; **Supplementary Figure S3B**).

**Table 1:** Growth parameters estimated in the *Bacillus* isolates in the CCB.

				Growth parameters			
				
	*rrn* copies	Isolate	Phylogenetic group	A	BBB (h)	μ_max_ (h^-1^)	*t*_d_ (h)
A	14	155B_5T	X. *B. cereus*	9.98	0.92	0.26	2.66
B	6	118_4C	XI. *B. sonorensis*	9.89	1.13	0.24	2.88
C	8	m3-18	II. *B.* sp. m2-34	9.78	2.89	0.24	2.88
D	9	m2-9	V. *B. coahuilensis*	7.95	1.08	0.22	3.15
E	10	m2-6	V. *B. coahuilensis*	7.68	1.45	0.21	3.30
F	12	112B_4D	XIV. *B. pumilus*	7.35	1.70	0.31	2.23
G	5	169A_5R	XIX. *Staphylococcus*	7.30	0.92	0.26	2.66
H	12	107_3D	IX. *B. marisflavi*	7.28	0.25	0.19	3.64
I	13	178_5C	XVI. *B. idriensis*	7.02	2.56	0.57	1.21
J	11	126_4D	X. *B. cereus*	6.69	2.78	0.23	3.01
K	7	152A_5R	I. *B. aquamaris*	6.48	0.22	0.16	4.33
L	8	44_1T	XVII. *B. horikoshii*	5.20	4.12	0.19	3.64
M	10	315_11T	IX. *B. marisflavi*	4.44	1.44	0.12	5.77
N	11	144b_14T	XIV. *B. pumilus*	3.67	2.79	0.11	6.30
O	6	108	XII. *B. atrophaeus*	3.38	2.06	0.08	8.66
P	11	127B_4D	XVII. *B. horikoshii*	3.22	1.70	0.09	7.70


*A = maximum biomass reached [*Ln*(*CFU/CFU*_(*t*__ =__0)_)]; BBB = lag phase; μ_max_ = maximum specific growth rate; *t*_*d*_ = doubling (generation) time.*

**FIGURE 3:**
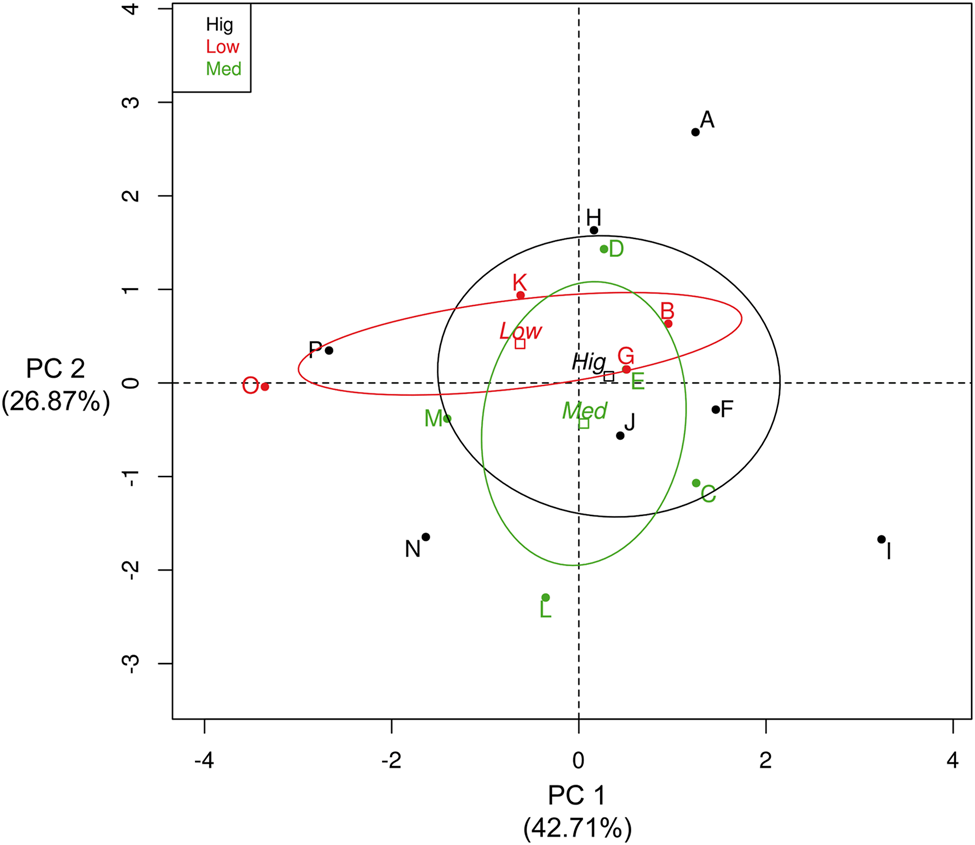
**Principal component analysis (PCA) of the growth parameters estimated in the *Bacillus* isolates from the CCB.** The names of isolates (letters, from A to P) were in agreement with the maximum biomass reached (*A*) in **Table 1.** Color labels represent the isolates of the arbitrary categorization of the number of copies of the *rrn* operon (upper left box), where red labels are the isolates with “low copy numbers” (five to seven), green labels are the isolates with “intermediate copy numbers” (8 to 10) and black labels are the isolates with “high copy numbers” (from 11 to 14).

Interestingly, an exploratory analysis showed no overall correlation between the number of copies of the *rrn* operon and the growth parameters estimated (**Supplementary Table S1**). However, an arbitrary categorization of this copy number (where “low” was from five to seven copies, “mid” was from eight to ten copies and “high” was from 11 to 14 copies) showed that the isolates with the fewest copies had lower levels of dispersion in their growth parameters (**Figure 3**). Principal component analysis (PCA) was performed to describe the influence of the various growth parameters in these categories in the isolates from the CCB (**Figure 3**). It seems that 42.71% of the variance was explained by Component 1, which was defined by doubling time (*t*_d_), maximum growth rate (μ_max_) and the maximum biomass reached (*A*). Component 2 explained 26.87% of the variance, and was principally defined by adaptation time (λ) and the *rrn* operon copies (**Supplementary Figure S4**). Meanwhile, isolates with the highest copy numbers showed greater dispersion, having the most extreme parameter values (**Table 1**). These results may indicate that the *rrn* operon copy number in the *Bacillus* from the CCB may be related to the integrated suite of growth dynamics parameters, but not exclusively to the growth rate. Then, the heterogeneity in the growth dynamics of the isolates of *Bacillus* from the CCB could be a response to the low availability of nutrients and the competitive cost that represents the high number of copies of the *rrn* operon.

### P and N Contents and N:P Ratios in the Exponential Phase

To assess potential eco-physiological implications associated with the oligotrophic conditions of the CCB regarding the genus *Bacillus*, biomass C, N, and P contents were estimated during the exponential phase of growth for all strains (**Supplementary Table S2**). All the isolates showed a relatively low but variable P (%) content (Mean = 0.496; *SD* = 0.616; Median = 0.258). While C (%) and N (%) content also showed high variability among the isolates [N (%): Mean = 7.14; *SD* = 5.61; Median = 5.49; C (%): Mean = 64.64; *SD* = 26.63; Median = 59.63]. Both the C:N ratio (Mean = 13.18; *SD* = 7.18; Median = 12.32) and the N:P ratio (Mean = 126.7; *SD* = 235.1; Median = 52.1) showed a considerable range (**Table 2**). Finally, the N:P ratios estimated in the *Bacillus* from the CCB were substantially higher than the ratios reported for other *Bacilli* (*B. subtilis* 10.6; Loladze and Elser, 2011).

**Table 2:** C:N and N:P ratio during the exponential phase of growth in the isolates of *Bacillus* from the CCB.

	*rrn* copies	Isolate	Phylogenetic group	C:N	N:P
A	14	155B_5T	X. *B. cereus*	4.94	965.38
B	6	118_4C	XI. *B. sonorensis*	19.28	10
C	8	m3-18	II. *B.* sp. m2-34	8.35	286.47
D	9	m2-9	V. *B. coahuilensis*	24.82	79.09
E	10	m2-6	V. *B. coahuilensis*	18.18	52.91
F	12	112B_4D	XIV. *B. pumilus*	18.77	194.61
G	5	169A_5R	XIX. *Staphylococcus*	5.75	25.83
H	12	107_3D	IX. *B. marisflavi*	8.87	19.55
I	13	178_5C	XVI. *B. idriensis*	17.85	40.67
J	11	126_4D	X. *B. cereus*	24.73	67.41
K	7	152A_5R	I. *B. aquamaris*	0.62	72.58
L	8	44_1T	XVII. *B. horikoshii*	13.79	4.61
M	10	315_11T	IX. *B. marisflavi*	16.7	82.72
N	11	144b_14T	XIV. *B. pumilus*	6.59	50.75
O	6	108	XII. *B. atrophaeus*	10.77	23.6
P	11	127B_4D	XVII. *B. horikoshii*	10.85	51.32
			Mean ± *SD*	13.18 ± 7.18	126.72 ± 235.13


**FIGURE 4:**
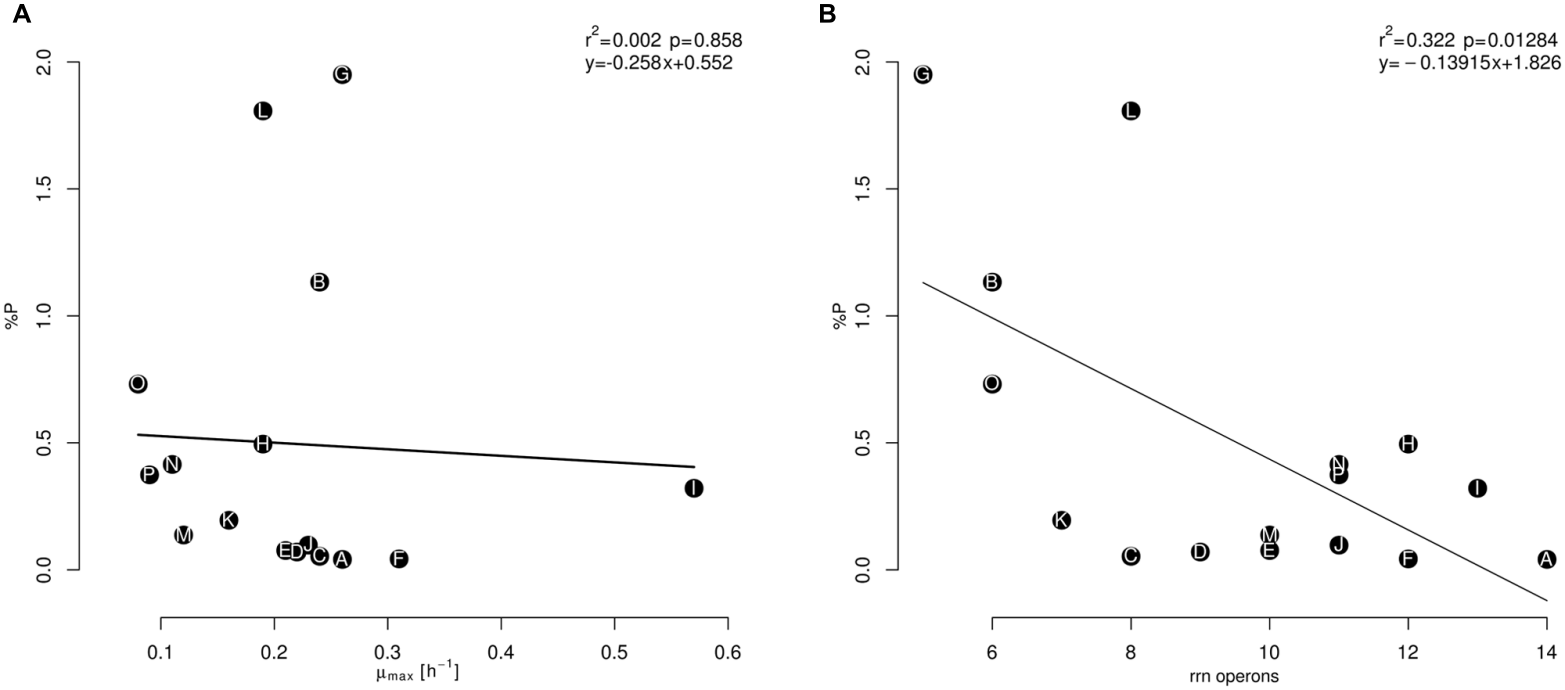
**Relationship between the maximum specific growth rate (μ_max_) **(A)** and the number of copies of the *rrn* operon **(B)**, along P (%) content, during the exponential phase.** The isolates names (letters, from A to P) are within the circles in black and were in agreement with the maximum biomass reached **(A)** in **Table 1.**

Despite the growth rate not being related to cellular P content (**Figure 4A**), the correlation between *rrn* operon copy number and P (%) content is negative and significant (**Figure 4B**). Isolate G, with a low copy number (five copies; *Staphylococcus*), had the highest cellular content of both elements. In addition, isolates B and O, with six *rrn* operon copies, had high P-content levels (*B. sonorensis* and *B. atrophaeus*, respectively). Meanwhile, isolate A (14 *rrn* operon copies; *B. cereus*) had the lowest P content of all the isolates analyzed; this isolate also had the second-highest N content. The isolates D and E (*B. coahuilensis*; nine and ten *rrn* operon copies, respectively) showed low P contents and had the lowest N content. Moreover, the previous isolates with low *rrn* operon copies (B, G, and O) showed low N:P ratios, while isolate A, with a high number of *rrn* operon copies had the highest estimated N:P ratio. In addition, the two isolates related to *B. coahuilensis* showed intermediate values of this elemental ratio (**Table 2**). These results were not consistent with the GRH and it seems that the isolates with low number of copies of the *rrn* operon may cope better in this oligotrophy of the CCB; although the number of operon copies quantified is high in comparison with other bacterial groups that live in other oligotrophic environments (e.g., cyanobacteria; Fegatella et al., 1998).

## Discussion

The general objectives of this work were to assess the variability of a particular ecological trait, the *rrn* operon copy number in *Bacillus* strains isolated from extremely oligotrophic aquatic ecosystems, and to evaluate whether there is any association between the variation of this trait and strain physiology. We analyzed 71 isolates of this ecosystem and found considerable variation in the ribosomal operon copy number with a tendency toward an intermediate number of *rrn* operon copies. We also documented variation in growth rate dynamics and elemental composition. While we did observe physiological associations consistent with the GRH (e.g., the isolate with the slower-growth showed the lowest P content, and a high ratio of N:P). However, there were no consistent associations between copy number and growth parameters. Instead, it is likely that a variety of genomic strategies beyond variation in *rrn* copy number are employed to modulate growth in this clade of *Bacillus*, potentially allowing for their coexistence in different niches.

### Phylogenetic Clustering of *Bacillus* Isolates

Our phylogenetic reconstruction showed a large diversity of species with isolates obtained from different habitats generally widespread in the tree. This suggests that each sampling site, as well as the overall system, contains several coexisting taxa. The microbial diversity in the Churince ecosystem has been deeply documented, particularly its *Bacillus* population, yielding 55 thermo-resistant strains and several extremely halotolerant strains (Cerritos et al., 2010). Moreover, endemic *Bacillus* strains have been described and genotyped. For instance, the genomes of *B. coahuilensis* and *Bacillus* m3-13 show several interesting low-nutrient adaptations (Alcaraz et al., 2008, 2010), as well as an ancient ancestry (Moreno-Letelier et al., 2011). Further sampling of pond sediments in Churince led to a demonstration of how antagonistic interactions between the *Bacilli* contribute to the large observed diversity, while maintaining a large local differentiation via either resistance or avoidance as in a paper-rock-scissors model (Pérez-Gutiérrez et al., 2013; Aguirre-von-Wobeser et al., 2014).

Most of our isolates came from Churince, whose large terminal lake is now mostly dry due to water overexploitation (Souza et al., 2006, 2012). Interestingly, several times during this sampling period, we recovered the same phylogroups in the sediments in the same sampling sites. It has been argued that sediment is the “native” habitat of *Bacillus* in the CCB because many phylogenetic groups coexist there and can be recovered consistently at different sampling times (Pérez-Gutiérrez et al., 2013). Our results support what has been observed previously: the considerable levels of bacterial diversity in this basin are the outcome of complex biotic interactions within the community, CCB’s ancient geological history, low nutrient availability, and considerable spatial and seasonal variability in environmental conditions (Souza et al., 2012).

### Variation in *rrn* Operon Numbers

We found no simple answer regarding variations in *rrn* operon copy number as a response on the part of bacteria in a low-nutrient environment. This was somewhat unexpected because *rrn* operon copy number is a well-studied functional trait that has been reported to be associated with bacterial lifestyle and represents an ecological strategy for nutrient use (Klappenbach et al., 2000; Stevenson and Schmidt, 2004; Green et al., 2008). A variety of studies have quantified the copy number of this operon in strains isolated from environmental samples, including some for which some *Bacillus* strains were analyzed (Klappenbach et al., 2000; Shrestha et al., 2007; Vieria-Silva and Rocha, 2015). However, no previous studies have focused on *rrn* operon copy number in such a diverse group of coexisting species within the genus *Bacillus*, much less in a shared environment with extremely low nutrient availability.

Previous work has described the genomic properties of some *Bacillus* isolated from the CCB (Alcaraz et al., 2008, 2010). For example, the genome analysis of *B. coahuilensis* documented nine *rrn* operon copies (Alcaraz et al., 2010; unpublished results). However, in our analysis, we quantified eight copies, perhaps because the differences in size among operons were too small to be detected via the pulse field method. Nevertheless, such discrepancies are common when these types of data are compared (Vishnivetskaya et al., 2009). *Bacillus* groups isolated from the CCB showed from six to 14 *rrn* operon copies, which is consistent with previous quantifications of the *rrn* operon (from six to 15 copies reported in *rrn*DB, Stoddard et al., 2015). This range of operon copies is not what would be expected for isolates from the CCB because the idea is that they should match a slower (i.e., “K-selected”) life history in an oligotrophic environment such as the CCB. This could be the case for all of the CCB isolates related to *B. sonorensis*, a soil strain that was first described in a desert sample with similar environmental conditions to those of the CCB (Palmisano et al., 2001; Souza et al., 2006, 2012). To evaluate this further, we compared our results with data from the *rrnDB* database. *B. atrophaeus* strain 1942 has seven *rrn* operon copies, but as mentioned above, in our analysis, we found a related strain with six copies. The *rrn*DB *B. subtilis* strains from the database showed from eight to ten copies; in our analysis, we observed a wider range (seven to eleven copies). On the other hand, seven copies of the *rrn* operon were observed in the *B. pumilus* genome, while *B. pumilus* relatives isolated in the CCB have eight, eleven, and twelve copies. Such intraspecific variability was evaluated by Acinas et al., (2004) for different bacterial genomes. They documented three species of *Bacillus* that had normal variation from one to three copies. Subsequent analysis with a larger number of *Bacillus* genomes showed similar variability (Rastogi et al., 2009). Accordingly, the CCB’s closely related, *pumilus*-like *Bacillus* showed a similar level of intraspecific variation, from one to four copies. Overall, the number of copies of the *rrn* operon in the genus *Bacillus* at the CCB shows considerable variability, but it does not show evidence of any clear directional change from previously published values for various taxa. This could be due to the fact that very large ranges of taxa within the *Bacilli* are being selected for a wide variety of responses to cope with the oligotrophic environment. For example, while some save P in their ribosomes with a slow growth rate (“K strategists”), others may maintain a high growth capacity (“r strategists”) that is compensated by other tactics, such as phospholipid to sulfolipid substitution, the presence of high-affinity P-uptake systems, and small genomes (Alcaraz et al., 2008; Moreno-Letelier et al., 2011).

### Growth Parameters and *rrn* Operon Copy Number

The analysis of bacterial growth is considered to be an important tool in understanding and characterizing an organism because it describes potential bacterial response to changes in environmental conditions, as well as ecological responses to other microorganisms (Monod, 1949; Neidhardt, 1999). As mentioned in the results section, the growth dynamics of *Bacillus* inhabitants of the CCB showed a high degree of variability in various parameters. This heterogeneity in growth rate has been previously observed for environmental strains with different numbers of *rrn* operons (Dethlefsen and Schmidt, 2007). In addition, several of the estimated parameters were similar to those described in other *Bacillus* strains under extreme experimental growth conditions (**Supplementary Table S3**); however, many of these previous studies were performed with model or economically important species. Previous work has considered the ecological importance of the *rrn* operon copy numbers in bacterial adaptation to different environmental conditions (Elser et al., 2000; Klappenbach et al., 2000; Shrestha et al., 2007; Green et al., 2008). Variation in the number of *rrn* operon copies is potentially related to the bacterial growth rate because of the need to sustain high levels of rRNA synthesis (Codon et al., 1995; Nanamiya et al., 2010; Yano et al., 2013). However, in our analysis, *rrn* operon copy number was not correlated with the estimated growth rate parameters. Instead, the growth dynamics of the *Bacillus* from the CCB may be dictated by a combination of different physiological responses that are uncoupled from the *rrn* operon copies, such as differences in transcription rates or intra-cellular allocation processes.

Among the various growth dynamics of the strains from the CCB, we observed not only that many parameters were out of range when compared with those estimated for other previously studied *Bacillus* but also that they presented high variability that at first inspection, does not seem to relate either to evolutionary history, isolation site, or *rrn* operon copy number. We hypothesize that the oligotrophic condition in the CCB may have had a significant effect on the *Bacillus* growth dynamics that contribute to this variability. For example, long adaptation times are related to stressful conditions affecting the speed of bacterial growth, like limited nutrient availability (Chorin et al., 1997; Schaechter, 2006; Antolinos et al., 2011, 2012; Bren et al., 2013). Furthermore, estimates of maximum biomass reached can be quite variable because of the influence of overall nutrient availability and sensitivity to the waste material that accumulates during the exponential phase (Buchanan et al., 1997; Chorin et al., 1997). In addition, long doubling times are characteristic of bacteria in oligotrophic environments (Vieria-Silva and Rocha, 2015). Indeed, bacteria from other ecosystems that are extremely limited in terms of nutrients can achieve generation times of thousands of years (Jørgensen and Boetius, 2007; Labonté et al., 2015). Thus, the bacterial growth properties of *Bacillus* that live in the extremely oligotrophic ecosystems of the CCB likely involve a complex response to environmental and nutritional conditions acting in concert with genomic potential.

### *rrn* Operon Copy Number and Phosphorus Availability in the CCB

As previously mentioned, ecosystems in the CCB are characterized by very low P availability in water, soil, and sediments (Elser et al., 2005; Peimbert et al., 2012), a condition that makes its high variation in *rrn* operon copy number somewhat surprising because we expected that such habitats would be dominated by taxa with low operon copy numbers. Previous work has suggested that *rrn* operon copy number is related to nutrient availability and especially with P because the copy number is linked to the growth rate, which is associated with the production of P-rich *rrn* (Elser, 2003; Jeyasingh and Weider, 2007). Indeed, it has been shown that bacteria that live in oligotrophic environments do tend to have very low *rrn* operon copy numbers (< two copies; Fegatella et al., 1998; Strehl et al., 1999; Lauro et al., 2009). In theory, multiple copies could allow a growth rate advantage when resources are abundant but would impose a competitive cost when resources are limited due to the costs of the over-production of *rrn* (Weider et al., 2005; Dethlefsen and Schmidt, 2007; Jeyasingh and Weider, 2007). While we detected many *Bacillus* isolates with six *rrn* operon copies, even this number is relatively high in comparison with other bacterial lineages that live under low nutrient conditions (Fegatella et al., 1998; Strehl et al., 1999; Lauro et al., 2009). Thus, it seems that the number of copies of the *rrn* operon is a functional trait related with the evolutionary history of the genus *Bacillus*, and defines its ecological versatility and adaptability to different environmental conditions (Klappenbach et al., 2000; Feldgarden et al., 2003; Stevenson and Schmidt, 2004; Connor et al., 2010).

A simple explanation of this incongruity is that the genus *Bacillus* does not pay for the full cost of high copy number under nutrient limitations due to its ability to escape from scarcity by forming spores and then germinating under better conditions. Indeed, stress response capacity has been shown to be related to *rrn* operon multiplicity (Nanamiya et al., 2010; Yano et al., 2013). However, spore formation in *Bacillus* of the CCB is not a given, due to the loss of many of the genes of the spore-forming complex in the sequenced genomes (Alcaraz et al., 2010) and the difficulty of obtaining spores experimentally in all the isolated strains. Nevertheless, *B. coahuilensis* m4-4 and *B.* sp. m3-13 have eight and nine *rrn* operon copies, respectively, like other spore-forming *Bacilli* (*B. halodurans* C-125, eight *rrn* operon copies; *B. amyloliquefaciens* CC178, 9 *rrn* operon copies). Thus, the variability in the *rrn* operon copy number could reflect broader life history strategies in which each taxa uses different sets of resources or inhabits distinct microhabitats in structured sediments, potentially decreasing competition. In agreement with this view, various bacterial communities have been shown to have similar functional heterogeneity regarding other important ecological traits (Martínez-Alonso et al., 2004; Giovannoni and Stingl, 2005; Martiny et al., 2006). Additional work is needed to understand the importance of conserving high *rrn* operon copy numbers in CCB *Bacillus*.

Our overall findings about diversity in *Bacillus rrn* operon number in the CCB do not seem to conform to the broader context of the GRH (Elser et al., 2000; Elser, 2006). For example, isolate A (*B. cereus*) had the highest number of copies of the *rrn* operon (14) and also had the slowest growth rate and highest N:P ratio (965) when growing on media produced using the CCB’s natural waters. Furthermore, several isolates with low *rrn* operon numbers showed some of the highest P-content values, contrary to the GRH. However, the high N:P ratio and slow growth rate of isolate A is consistent with the development of severe P limitations for this high-copy-number strain, resulting in its high biomass N:P ratio. Then, it seems that the isolates with lower number of copies of the *rrn* operon may cope better in this oligotrophy of the CCB. To more effectively test the genetic dimension of the GRH, each strain would need to be raised under optimal conditions at its genetically constrained maximal growth rate. Indeed, this inference is supported by the higher dispersion of growth parameters seen for high-copy-number strains. That is, low-copy-number strains may have a limited range of growth variation, regardless of media, while high-copy-number strains may have a considerable range of growth, depending on whether or not the environment is well-matched to their needs. Another possible explanation for the decoupling of *rrn* operon number from growth rate and stoichiometric properties is that CCB *Bacillus* are selected for fine-tuned signaling with resource supplies or for variation in the rates of rRNA genes expression, thus disconnecting copy number from RNA production and growth rate (i.e., low-copy-strains may have high levels of transcription for each copy, while high-copy strains may more stringently express each copy). In any case, our data do not provide a clear resolution regarding the validity of the genetic components of the GRH within the *Bacillus* of the CCB. It is possible that the consideration of a broader range of bacterial taxa, as well as the more extensive testing of growth conditions, are needed in order to more rigorously test the GRH in the microbial realm, using approaches that can overcome the possible impacts of physiological conditions, phylogenetic inertia, and taxon-specific lifecycle strategies (e.g., sporulation) in terms of confounding the interpretations.

We suggest that the growth patterns described in the *Bacillus* isolates from the CCB, as well as the high variability in *rrn* operon copy number, represent ecological strategies that allow them to persist in this oligotrophic ecosystem. Analogous strategies have been described in other organisms whose growth parameters are also affected by various environmental and biotic factors (Pianka, 1970; Page, 2002; Lipowsky et al., 2012). Variable lag phases may help in adapting to changing environmental conditions to reach optimum growth with long generation times (Crooks, 2005; Wangen and Webster, 2006; Daehler, 2009). We also note that the retardation of growth is a common result of intense interaction with other organisms, as well as stressful conditions (Gao et al., 2013; Tsugama et al., 2014). To date, these ecological strategies in bacteria have been largely related only to nutrient availability (Klappenbach et al., 2000; Fierer et al., 2007; Shrestha et al., 2007). However, the intensity of direct inter-specific interactions (such as chemical antagonisms) can also establish coexistence, and these interactions are known to be particularly intense in the CCB (Souza et al., 2012; Pérez-Gutiérrez et al., 2013; Aguirre-von-Wobeser et al., 2014). These inferences suggest a bet-hedging strategy on the part of CCB bacteria in which expression of RNA genes is tightly controlled due to low P-conditions (reflected in their overall low P content and high N:P ratio), but when chemical antagonism is successful, resources suddenly arrive, and the *rrn* operons are activated to grow rapidly under the nutrient bounty.

The main results of our work indicate that the *rrn* operon copy number exhibits considerable variation among field-isolated *Bacilli* and that considerable variation also exist in their growth properties and chemical composition. However, *rrn* operon copy number appears to be largely uncoupled from growth and chemical properties in this clade. Further investigation is needed to understand the ecological and physiological importance of this *rrn* operon variability, as mediated by gene transcriptional regulation, and its influence on ribosome and protein content and thus N:P stoichiometry (Gourse et al., 1996; Fegatella and Cavicchioli, 2000; Dethlefsen and Schmidt, 2007; Scott et al., 2010; Piir et al., 2011). It may be that the extreme oligotrophic conditions in the CCB have imposed important physiological constraints on resource allocation and growth rate, as well as the expression of the rRNA genes, and thus, the rate of production of ribosomes per *rrn* operon copy differs considerably among strains. More detailed studies, including competition experiments involving *Bacillus* strains isolated from different CCB environments and subject to various environmental limitations (such as differences in nutrient supply concentrations, ratios, and supply schedules), may be needed to identify the ecological and evolutionary significance of *rrn* operon copy number variation among microbes in the habitats of Cuatro Ciénegas and similar nutrient-deficient habitats.

## Author Contributions

JV-A: Primary author, experimental design, amplification and analysis of genetic material, PFGE, analysis of data. LE-F: Experimental design, analysis of data. GD-S: PFGE standardization, analysis of data. PM-Z: Growth curves standardization, parameter estimations. JG-P: Statistical analysis. JL: Cell contents of phosphorus (P) and nitrogen (N). JE: Experimental design, analysis of data. GO-A: Experimental design, analysis of data. VS: Group leader, experimental design, analysis of data.

## Conflict of Interest Statement

The authors declare that the research was conducted in the absence of any commercial or financial relationships that could be construed as a potential conflict of interest.

## Supplementary Material

**FIGURE S1 | Growth conditions assays and Gompertz growth curves of the *Bacillus* isolates from the CCB.** **(A)** Different nutritional conditions were analyzed to define the optimum conditions and thus describe the growth dynamics of the *Bacillus*: A = Marine broth (Difco^TM^ 2216); B = CCB water; C = CCB water + P (KH_2_PO_4_, 5 g per liter); and D = CCB water + N (tryptone, 5 g per liter). Growth was quantified via culture turbidity (OD_600_; BioPhotometer Plus, Eppendorf) after 12 h of growth, and the data were normalized to define the optimal conditions for growth. **(B)** Growth curves were described after linear regression analysis according to the Gompertz equation. Isolate names were in agreement with the maximum growth reached (*A*), followed by the number of copies of the *rrn* operon for every isolate. Growth curves were plotted with the data of the plate count assays [*Ln*(*CFU/CFU*_(*t*__ =__0)_)].

**FIGURE S2 | rRNA operon copy number in the genus *Bacillus* isolated in the CCB.** For every isolate, the separation of fragments with PFGE is shown on the left and Southern-blot confirmation is shown on the right. At the bottom of the figure is the total number of rRNA operon copies quantified. The red asterisk represents the fragments identified with hybridization analysis.

**FIGURE S3 | Relationship between the growth parameters estimated in the *Bacillus* isolates from the CCB.** **(A)** λ vs μ_max_ and, **(B)** μ_max_ vs A. Isolate names (letters, from A to P) are within the circles in black and were in agreement with the maximum biomass reached **(A)** in **Table 1.** A = maximum biomass reached [Ln(CFU/CFU(_t=0_))]; λ = lag phase; μ_max_ = maximum specific growth rate.

**FIGURE S4 | Relative contribution (%) per variable in the PCA of the growth parameters estimated in the *Bacillus* isolates from the CCB.** A = maximum growth reached; λ = lag phase; *t*_d_ = doubling time; and μ_max_ = maximum specific growth rate.
