# Supplementary figures and images for: Variability of rRNA Operon Copy Number and Growth Rate Dynamics of Bacillus Isolated from an Extremely Oligotrophic Aquatic Ecosystem
Source: Front Microbiol. 2016 Jan 5;6:1486. doi: 10.3389/fmicb.2015.01486 (PMC4700252; doi:10.3389/fmicb.2015.01486)

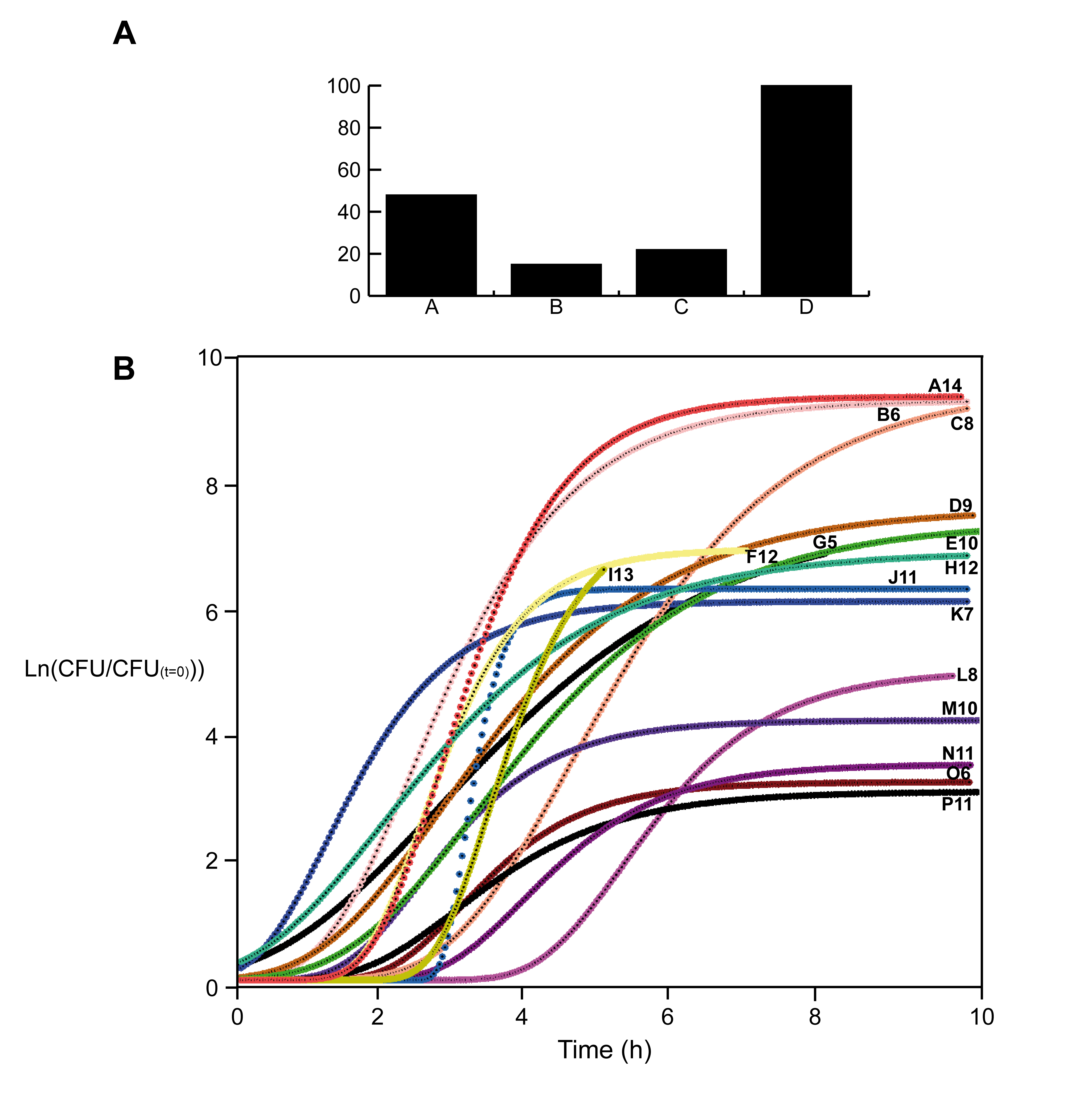

Supplement: Supplementary file 1 [file Image_1.TIF]

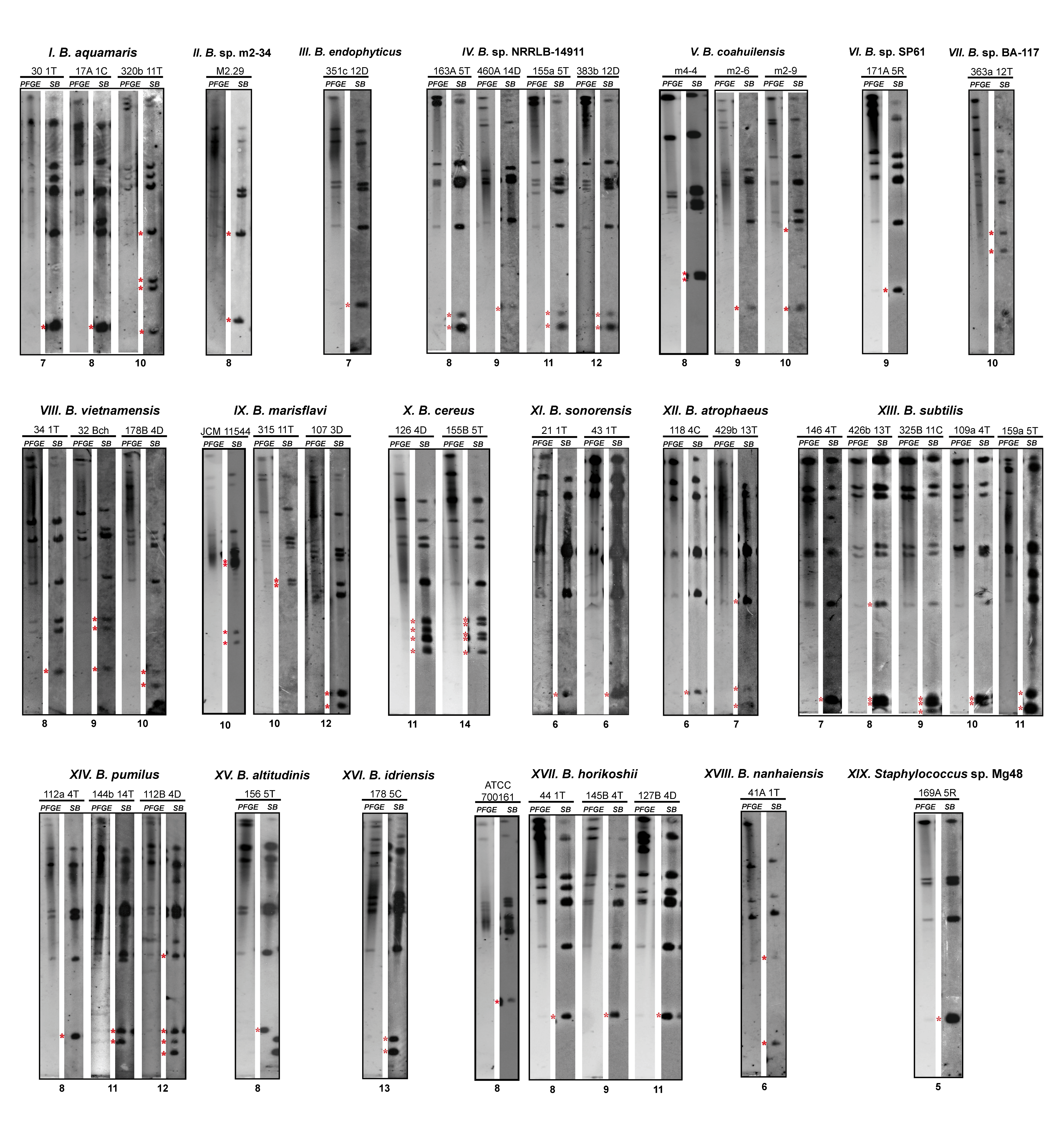

Supplement: Supplementary file 2 [file Image_2.TIF]

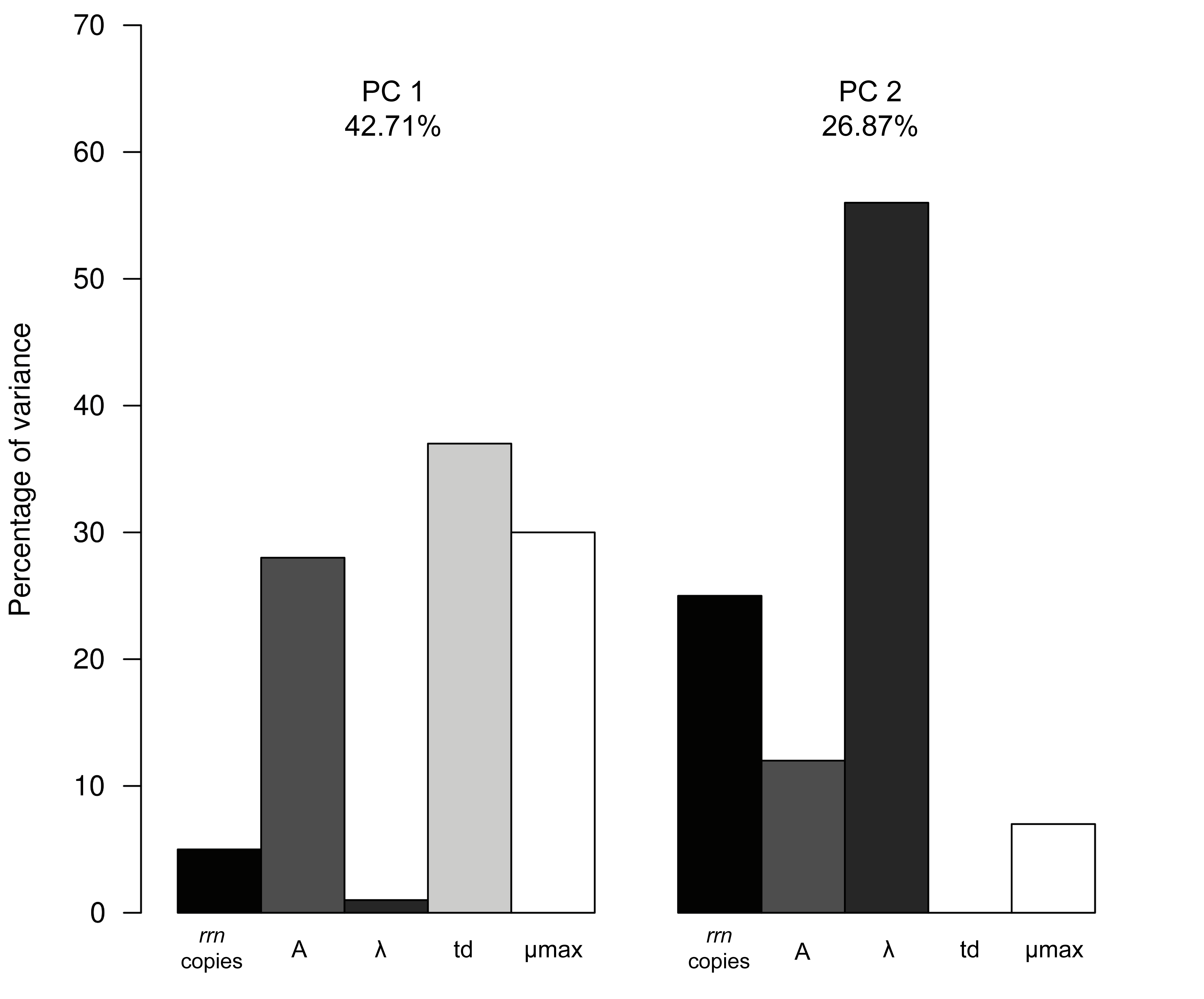

Supplement: Supplementary file 4 [file Image_4.TIF]
