# Supplementary material for: Variability of rRNA Operon Copy Number and Growth Rate Dynamics of Bacillus Isolated from an Extremely Oligotrophic Aquatic Ecosystem
Source: Front Microbiol. 2016 Jan 5;6:1486. doi: 10.3389/fmicb.2015.01486 (PMC4700252; doi:10.3389/fmicb.2015.01486)
Supplement: Supplementary file 5 [file Table_1.DOCX]

Supplementary Material

**Variability of rRNA operon copy number and growth rate dynamics of *Bacillus* isolated from an extremely oligotrophic aquatic ecosystem**

**Jorge A. Valdivia-Anistro^1^, Luis E. Eguiarte^1^, Gabriela Delgado^2^, Pedro Márquez-Zacarías^3^, Jaime Gasca-Pineda^1^, Jennifer Learned^4^, James J. Elser^4^, Gabriela Olmedo^5^ and Valeria Souza^1*^**

*** Correspondence:** Valeria Souza, Laboratorio de Evolución Molecular y Experimental, Instituto de Ecología, Departamento de Ecología Evolutiva, Instituto de Ecología, Universidad Nacional Autónoma de México, AP 70-275, Coyoacán, 04510, México DF, México.

souza@unam.mx

**Table 1S.** Pearson correlation coefficient (*r*) of the growth parameters estimated in the *Bacillus* isolates from the CCB.

|  |  | **1** | **2** | **3** | **4** |
| --- | --- | --- | --- | --- | --- |
| **1** | *rrn* copies |  |  |  |  |
| **2** | A | 0.043 |  |  |  |
| **3** | λ | 0.039 | -0.284 |  |  |
| **4** | μ_max_ | 0.337 | 0.511^*^ | 0.099 |  |
| **5** | t_d_ | -0.213 | -0.804^***^ | 0.046 | -0.804^***^ |
| **P<0.05 **P<0.01 ***P<0.001* | | | | | |
