# Supplementary material for: Variability of rRNA Operon Copy Number and Growth Rate Dynamics of Bacillus Isolated from an Extremely Oligotrophic Aquatic Ecosystem
Source: Front Microbiol. 2016 Jan 5;6:1486. doi: 10.3389/fmicb.2015.01486 (PMC4700252; doi:10.3389/fmicb.2015.01486)
Supplement: Supplementary file 6 [file Table_2.DOCX]

Supplementary Material

**Variability of rRNA operon copy number and growth rate dynamics of *Bacillus* isolated from an extremely oligotrophic aquatic ecosystem**

**Jorge A. Valdivia-Anistro^1^, Luis E. Eguiarte^1^, Gabriela Delgado^2^, Pedro Márquez-Zacarías^3^, Jaime Gasca-Pineda^1^, Jennifer Learned^4^, James J. Elser^4^, Gabriela Olmedo^5^ and Valeria Souza^1*^**

*** Correspondence:** Valeria Souza, Laboratorio de Evolución Molecular y Experimental, Instituto de Ecología, Departamento de Ecología Evolutiva, Instituto de Ecología, Universidad Nacional Autónoma de México, AP 70-275, Coyoacán, 04510, México DF, México.

souza@unam.mx

**Table 2S.** Carbon (C), nitrogen (N) and phosphorus (P) content during the exponential phase of growth in the isolates of *Bacillus* from the CCB.

|  | ***rrn* copies** | **Isolate** | **Phylogenetic group** | **%C** | **%N** | **%P** |
| --- | --- | --- | --- | --- | --- | --- |
| A | 14 | 155B_5T | X. *B. cereus* | 74.61 | 17.57 | 0.041 |
| B | 6 | 118_4C | XI. *B. sonorensis* | 84.61 | 5.11 | 1.133 |
| C | 8 | m3-18 | II. *B.* sp. m2-34 | 48.97 | 6.83 | 0.053 |
| D | 9 | m2-9 | V. *B. coahuilensis* | 51.92 | 2.44 | 0.07 |
| E | 10 | m2-6 | V. *B. coahuilensis* | 27.76 | 1.79 | 0.076 |
| F | 12 | 112B_4D | XIV. *B. pumilus* | 57.07 | 3.55 | 0.043 |
| G | 5 | 169A_5R | XIX. *Staphylococcus* | 112.3 | 22.75 | 1.95 |
| H | 12 | 107_3D | IX. *B. marisflavi* | 33.26 | 4.35 | 0.495 |
| I | 13 | 178_5C | XVI. *B. idriensis* | 89.92 | 5.87 | 0.321 |
| J | 11 | 126_4D | X. *B. cereus* | 62.19 | 2.93 | 0.097 |
| K | 7 | 152A_5R | I. *B. aquamaris* | 3.43 | 6.3 | 0.195 |
| L | 8 | 44_1T | XVII. *B. horikoshii* | 44.61 | 3.77 | 1.807 |
| M | 10 | 315_11T | IX. *B. marisflavi* | 73.07 | 5.1 | 0.137 |
| N | 11 | 144b_14T | XIV. *B. pumilus* | 53.46 | 9.45 | 0.415 |
| O | 6 | 108 | XII. *B. atrophaeus* | 72 | 7.81 | 0.731 |
| P | 11 | 127B_4D | XVII. *B. horikoshii* | 81.03 | 8.69 | 0.374 |
|  |  |  | Mean ± Std. dev. | 60.64 ± 26.63 | 7.14 ± 5.61 | 0.496 ± 0.616 |
