# Supplementary material for: Variability of rRNA Operon Copy Number and Growth Rate Dynamics of Bacillus Isolated from an Extremely Oligotrophic Aquatic Ecosystem
Source: Front Microbiol. 2016 Jan 5;6:1486. doi: 10.3389/fmicb.2015.01486 (PMC4700252; doi:10.3389/fmicb.2015.01486)
Supplement: Supplementary file 7 [file Table_3.DOCX]

Supplementary Material

**Variability of rRNA operon copy number and growth rate dynamics of *Bacillus* isolated from an extremely oligotrophic aquatic ecosystem**

**Jorge A. Valdivia-Anistro^1^, Luis E. Eguiarte^1^, Gabriela Delgado^2^, Pedro Márquez-Zacarías^3^, Jaime Gasca-Pineda^1^, Jennifer Learned^4^, James J. Elser^4^, Gabriela Olmedo^5^ and Valeria Souza^1*^**

*** Correspondence:** Valeria Souza, Laboratorio de Evolución Molecular y Experimental, Instituto de Ecología, Departamento de Ecología Evolutiva, Instituto de Ecología, Universidad Nacional Autónoma de México, AP 70-275, Coyoacán, 04510, México DF, México.

souza@unam.mx

**Table 3S.** Growth parameters estimated in different *Bacillus* strains*.*

|  |  | **Growth parameters** | | | | |  |  |
| --- | --- | --- | --- | --- | --- | --- | --- | --- |
| **Specie** | **Strain** | **λ**  **(h)** | **μ**  **(h^-1^)** | **t_d_**  **(h)** | **G_t_**  **(h)** | **Temp.**  **(ºC)** | **Comments** | **Reference** |
| *B. mycoides* |  | - | - | - | 0.48 | 35 | Tryptic meat broth | In Powell, 1956 |
| *B. cereus* | 569/H | - | 3.27 | - |  | 35 | Measurements of bacterial length among divisions | Collins  & Richmond, 1962 |
| *B. cereus* | B687 | - | 2.8 | - |  | 42 | Maximum growth rate temperature* | Warth, 1978 |
| *B. cereus* subsp. *mycoides* |  | - | 1.2 | - |  | 25 |  |  |
| *B. subtilis* | P | - | 3.2 | - |  | 46 |  |  |
| *B. subtilis* | 168 | - | 2.9 | - |  | 46 |  |  |
| *B. subtilis* | B692 | - | 2.8 | - |  | 46 |  |  |
| *B. subtilis* subsp. *niger* |  | - | 2.5 | - |  | 43 |  |  |
| *B. subtilis* |  | - | - | - |  | 7  to  43 | Predictions to obtain the minimum, maximum and optimum growth temperature* | Ratkowsky et al., 1983 |
| *B. subtilis* | 168/S | - | 0.0058^†^ | 2.0 |  | 35 | Growth rate calculated with the Collins-Richmont principle modified | Burdett et al., 1986 |
| *B. cereus* | F2797/87, F3351/87, F3752A/86, F196/73, F3748/75 and B-6/Ac | - | - | 0.25 to 3.77 |  | 10  to  30 | Predictions of doubling time in different foods and growth conditions. Mixed inoculum. | Shuterland et al., 1996 |
| *B. cereus* |  | - | - | - |  | 7  to  30 | Predictions at different temperature, pH and water activity | Chorin et al., 1997 |
| *B. stearothermophilus* | ATCC 12980 | - | 0.916 | - |  | 45  to 65 | Salty carrot medium | Ng & Schaffner, 1997 |
| *B. subtilis* | ATCC 6051 | 9-10 | 0.31-0.33 | - |  | 37 | Space flight cultures | Kacena et al., 1999 |
| *B. cereus* |  | ~20  to  180 | ~0.1  to  0.35 | - |  | 5, 7, 9, 11 and  13 | Growth in pasteurised milk | Valík et al., 2003 |
| *B. mojavensis* | ATCC 39307 | - | 0.6-1-3 | - |  | 37 | Use of deoxyribonucleosides or DNA to growth under anaerobic conditions | Folmsbee et al., 2004 |
| *B. cereus* |  | - | - | - |  | 2  to  48 | Isolated from different foods and grow at different temperature | Membré et al., 2005 |
| *B. cereus* | INRA-AVZ421 | 3.41  1.11 | 0.37  1.34 | -  - |  | 16  25 | Growth under stressful conditions | Antolinos et al., 2011 |
| *B. weihenstephanensis* | KBAB4 | 1.3  1.6  1.8 | 1.558  1.253  0.979 | -  -  - |  | 30 | Effects on growth parameters after shifts in temperature and water activity | Antolinos et al., 2012 |
| *B. subtilis* | TF8A | - | - | - |  | 37 | Nutrient influence in the stationary phase | Chubukow & Sauer, 2014 |

λ = Lag phase. *The groups similar to the CCB were only mentioned. ^†^ Specific growth rate (*k*) in doubling times per min. t_d_ = doubling time. G_t_ = generation time.
